# Post-simulation debriefing as a stepping stone to self-reflection and increased awareness — a qualitative study

**DOI:** 10.1186/s41077-024-00306-2

**Published:** 2024-08-13

**Authors:** Sissel Eikeland Husebø, Inger Åse Reierson, Anette Hansen, Hilde Solli

**Affiliations:** 1 Department of Quality and Health Technology, Faculty of Health Sciences, University of Stavanger, Post Box 8600, 4036 Stavanger, Norway; 2 Department of Nursing and Health Sciences, Faculty of Health and Social Sciences, Research Group Clinical Competence in Nursing Education, University of South-Eastern Norway, Post Box 4, 3199 Borre, Norway

**Keywords:** Debriefing, Facilitator, Focus group interviews, Nursing students, Reflection, Reflective practice, Simulation-based learning, Systematic text condensation, Qualitative study

## Abstract

**Background:**

The voice of the students should be engaged in simulation curriculum development. Involving the students in the development of debriefing strategies might result in a deeper understanding of learning. However, few studies have investigated the students’ perspectives on debriefing strategies. The aim of the study was to explore nursing students’ perspectives on the post-simulation debriefing.

**Methods:**

An explorative, descriptive design with a qualitative approach was used. Data were collected in December 2017 and May 2018 through focus group interviews with undergraduate nursing students in Norway immediately after a 2-day high-fidelity simulation course in the second year of their Bachelor of Nursing degree. Data were analysed using systematic text condensation.

**Results:**

Thirty-two nursing students participated in the study. The data analysis identified two main categories. The category ‘Facilitator as a catalyst for reflection’ illustrated the facilitator’s multifaceted and vital role in initiating and guiding the students’ reflection process in the debriefing. The category ‘A process towards increased awareness’ encompasses the students’ guided process of acquiring new insight into their professional development, and how they put parts together to see the wholeness in what was simulated.

**Conclusions:**

This study provides knowledge to facilitators regarding nursing students’ perspectives on facilitating reflection and learning during debriefing discussions. The facilitator’s multifaceted role in guiding the students’ reflections and their process of acquiring new insight into their professional development were identified as critical to learning during debriefing.

**Supplementary Information:**

The online version contains supplementary material available at 10.1186/s41077-024-00306-2.

## Background

Simulation-based learning (SBL) is an increasingly used learning platform in nursing education [1–4]. It allows students to mimic simple and complex clinical situations in a safe, structured and supported environment with reduced risk of harming patients in clinical practice [5]. SBL can be used to train life-threatening clinical scenarios without risk to patients or nursing students [6]. The lack of clinical facilitators and clinical placements supports the more extensive use of SBL in nursing education [7, 8].

When planning, implementing and evaluating different learning platforms, the students’ voices should be engaged in simulation curriculum development [9]. Involving the students in developing simulation learning platforms might result in a deeper understanding of learning, positively influence the relationship between students and teachers, and increase students’ motivation [10]. The post-simulation debriefing is described as the most crucial part of SBL [11–14], and nursing students perceive the debriefing as an approach to facilitate meaningful reflection and enhance learning [15]. Debriefing can be defined as a collaborative, formal, reflective process that takes place within the simulation learning activity [16]. The debriefing aims to facilitate the students’ reflective thinking and development of insight, enhance performance and transfer learning to clinical practice [17]. The debriefing allows the nursing students to explore their emotions, ask questions, reflect and analyse their own and peers’ performance, decisions and the simulation result [18].

Furthermore, the debriefing is essential to receive constructive feedback and reveal what is necessary for improvement and for enhancing knowledge transfer from the simulation into clinical situations [19, 20]. Previous research shows that adding video review during the debriefing process improved learners’ experience, attitude and performance, but it did not show its advantage over debriefing without video review on knowledge acquisition [21]. A review by Niu et al. [22] showed that video-assisted debriefing was more effective for nursing students’ experiences and critical thinking compared to debriefing without video review. Zhang et al. [23] explored nursing students’ perspectives on video-assisted debriefing. They found that video not only complemented the drawback of debriefing without video review by offering objective evidence but also improved their attitudes and behaviours through the experience of a myriad of emotions. The students also faced several challenges using video; for example, the camera did not capture important actions and video watching was too time-consuming.

Reflection in the debriefing is essential for learning [24] and plays a significant role in nursing students’ development of knowledge, clinical judgement and understanding [25, 26]. The debriefing comprises more than reflections on achieved results; it also comprises emotions and thoughts underlying what was said or done in the situation [19, 27]. Reflection can be described as an inner conversation that connects past experiences to the present and, potentially, the future. It involves contemplating our thinking and reasoning our thoughts, emotions and experiences. The thinking, feelings and actions included in the reflection process form the learning [25].

The role of the facilitator incorporates structuring the debriefing, facilitating a reflective and student-led conversation, and viewing multiple perspectives [28]. A systematic debriefing might support and assist the students in achieving the learning objectives. It requires a competent facilitator with knowledge and skills in the simulation subject and technicalities, the ability to provide appropriate feedback, debriefing and/or guided reflection [17], and experience from relevant clinical areas [29]. The Healthcare Simulation Standards of Best Practice™ (HSSOBP) Facilitation [30] criteria emphasize that the “facilitator who guides the debriefing is recommended to have specific skills and knowledge in simulation pedagogy” (p. 23). Previous research on the facilitator’s perception of the role emphasizes their ability to be attentive and adaptive to the emotional and cognitive responses of the students and create a safe learning environment, which is crucial for the students’ ability to reflect and learn [31, 32]. A concept analysis of student-centered reflection in debriefing concluded that by engaging in behaviours that promote reflection during debriefing, facilitators can improve clinical judgement, foster new understanding and promote behaviour changes, following SBL experiences [33]. Hall and Tori [34] identified the best practice guidelines for the debriefing phase of SBL. They found that assessment and training of the person who conducts the debriefing, along with the method and structure of the debriefing, could impact student learning.

The post-simulation debriefing in nursing education has been well-researched [35]. However, previous research often has examined the nursing students’ perspectives on the whole simulation process and not the debriefing alone [15]. Recent studies showed that SBL provoked students’ stress and anxiety about not having the proper knowledge and skills, being observed and judged, and getting feedback from peers and facilitators [36–38]. In these studies, the students expressed the importance of having a skilled and engaging facilitator who would be mindful of their emotional reactions. When learning something new, the students preferred facilitator-led debriefings because they trusted the facilitators’ knowledge and experiences in providing feedback. However, when reaching a particular level of proficiency, peer-led debriefing and feedback could be very useful because the facilitator’s presence could increase stress [23].

Knowledge of students’ perspectives on the facilitator’s role in the debriefing is essential because a non-satisfactory debriefing might result in decreased involvement, inaccurate learning and poor clinical judgement [34, 39]. Only a few studies have investigated how the facilitator’s role affects students’ ability to reflect, learn and achieve learning objectives [33]. In Coutinho et al.’s [40] study, the students perceived a structured debriefing as facilitating a closer and more empathic relationship between them and the facilitator. The facilitator helped them structure their thoughts and did not dwell on the mistakes but was open to questions and provided constructive feedback. Ko and Choi [37] found that the nursing students felt uncomfortable because the facilitators did not follow a standardized debriefing method, and they felt emotionally injured when receiving negative feedback from the facilitator during the debriefing. Nagle and Foli [26] found in their study of students’ experiences of reflection during debriefing that facilitators and peers’ engagement contributed to a supportive environment that promoted reflection on their own and peers’ actions in a collaborative group discussion. The findings also highlighted the facilitator’s guidance as important for discovering connections and remembering what to do or not in future clinical situations. Similarly, Nash and Harvey [41] found that for simulated learning to be meaningful from the student’s perspective, the facilitator played a fundamental role during the debriefing providing metacognitive guidance to assist students in contextualising learning. To summarize, little is known regarding nursing students’ perspective on the facilitator’s role in the debriefing. Such knowledge is pivotal for improving debriefing strategies.

## Aim

The study aimed to explore nursing students’ perspectives on the post-simulation debriefing.

Specifically, the research questions were:

1. How do the nursing students perceive the facilitator’s role in the debriefing?
2. How do the nursing students perceive the debriefing as a learning experience?

## Methods

### Design

This study had an explorative, descriptive design with a qualitative approach. This design is appropriate when knowledge of a phenomenon is scarce [42]. As in-depth knowledge of the debriefing from the students’ perspective is scarce, the chosen design was regarded as appropriate [43, 44]. Focus group interviews (FGIs) were determined as a suitable method for data collection. The interaction in an FGI, where the participants share and respond to each other’s thoughts, experiences and perceptions, might result in new viewpoints and generate rich and diverse data [45].

### Participants

Nursing students were recruited from a 3-year bachelor’s programme in nursing. All students had studied the same curriculum and achieved the same learning objectives prior to a mandatory SBL course. Before the students attended the mandatory SBL course, 137 were invited to participate in the study. Thirty-eight students gave written consent to participate.

### Setting

The study was carried out in a simulation centre at a Norwegian university. The SBL course was a mandatory preparation for Bachelor of Nursing students in their second year of education before clinical placements in medical and surgical units. The Norwegian language was used in the teaching of nursing education, and this also applies to SBL. The reflections in the debriefing and the FGI afterwards were in their mother tongue. Therefore, the students’ reflections provided a good description in purely linguistic terms.

The SBL scenarios focused on the acute deterioration of patients with medical or surgical conditions. The pre-briefing information was provided orally in class and written information via the students’ digital learning platform. This information included a video showing the functions of the high-fidelity simulators (HFSs), descriptions of the scenarios, including learning objectives and relevant literature, and a digital multiple-choice questionnaire with individual electronic feedback, which covered core knowledge associated with each scenario. Each simulation session lasted 85 min, comprising a briefing (15 min), the simulated scenario (15 min), watching a film and group reflection (15 min – Part 1) and the debriefing (40 min – Part 2). The students were divided into groups of seven to eleven. Each group participated in six different simulated scenarios within 2 days. Two students in each group participated in the simulated scenario as nurses, while the remaining students participated as observers. Their task was to observe the nurses’ actions according to a structured observation tool describing appropriate nursing interventions related to learning objectives for each scenario regarding airways, breathing, circulation, disability and exposure (A, B, C, D, E) [46], prioritisation, leadership and communication. Three HFSs were used. The software used automatically recorded the simulated scenario.

The debriefing consisted of two parts. In the first part, the two students who participated as nurses watched the video recording of their performance while the observing students discussed their observations in a separate room. In the second part, the students gathered for a facilitator-led debriefing driven by the observation tool and a standard debriefing that included a review of positive points, opportunities for improvement and advice on improving performance. The facilitator used three strategies: self-assessment, focused discussion and directive feedback or teaching. One facilitator guided each debriefing. All facilitators were registered nurses and university faculty, with completed facilitator courses and several years of experience as facilitators in nursing education.

The current study is part of a larger study exploring nursing students’ perspectives on the role of the facilitator in the briefing and the facilitator and operator in the simulated scenario in SBL [47–49]. This study concerns the debriefing.

### Data collection

Data collection was carried out in December 2017 (full-time students) and May 2018 (part-time students). A semi-structured interview guide (in Norwegian) was developed by the researchers (Additional file 1), all of whom were experienced in facilitating SBL and SBL research. Thirty-two of the 38 students who consented participated in FGI. Participants were divided into five FGIs (three with full-time and two with part-time students), four of which consisted of six to nine participants. Four students did not show up; therefore, one FGI had only two participants. As the use of small FGIs is supported by literature [50], the latter FGI was also included in the data analysis. The FGIs took place at the university immediately after the SBL courses ended. The interviews were audio recorded and lasted between 60 and 90 min.

### Analysis

A professional agency conducted a verbatim transcription of the FGIs. Systematic text condensation [44] was used to analyse the data. All authors contributed to the analysis process. The researchers were registered nurses with long experience as nursing teachers and facilitators in SBL, and they had extensive research experience using qualitative methodologies in nursing education and simulation. Three authors (SEH, IÅR, HS) were skilled facilitators in SBL.

The analysis was conducted in four steps. In the first step, all authors read the FGI transcripts to acquire a first impression of the whole and the identified preliminary themes. In the second step, meaning units related to the preliminary themes were identified and marked with codes. The codes were grouped into categories and sub-categories (drafted by HS). Before entering step 3, the grouping of codes and suggestions of categories and sub-categories were discussed and refined (by AH and HS). In the third step, code groups and subgroups were condensed and abstracted (by AH and HS). In the last step, the abstracted contents of the condensates were synthesized (by AH). A comparison between the synthesized text and the original transcripts took place to ensure that the synthesized text reflected the wholeness of the original transcripts. All authors have contributed to all analysis steps by reading all the FGIs and discussing meaning units, codes, sub-categories, main categories and quotations in several meetings. Throughout the analysis, the authors were reflexive through discussion, review and writing. This cooperation was essential to secure each stage in the analysis process [44]. An extract of the analysis is presented in Table 1.

**Table 1:** An extract of the analysis

Meaning units	Subcategory	Main category
The facilitators showed understanding for the lack of experience. They asked questions but did not push, rather explained. The facilitator asked good questions for reflection, which challenged us to think for ourselves	Balancing safety and challenges	Facilitator as a catalyst for reflection
The facilitator emphasized the positive and had a constructive way of giving feedback. We needed feedback on whether we understood and acted correctly. Gave useful feedback also on mistakes and potential for improvement	Providing constructive feedback	
The facilitator described variations using theory and by sharing own experiences, which was very useful. It was instructive when the things that happened were linked to knowledge and when our actions were put into context with possible outcomes. Gave examples that showed us options for action. There is not only one solution, but there are many ways to act	Facilitating the discovery of coherence and alternative options	
The facilitator had a guiding role. The facilitator took control and ensured we got through everything we had to, and everyone got to talk. The facilitator gave just the right amount of time for reflection	Providing structure as a prerequisite for dialogue	

### Reflexivity

All authors adopted a reflexive attitude to discuss their initial impressions of the data concerning their interests and biases. They considered their professional roles as faculty in nursing education and how these might affect their initial impression of the data. To minimize the potential influence on nursing students’ responses, all FGIs, except two (the first author participated as a co-moderator), were conducted by four faculty at the university, none of whom facilitated the simulation course. All were registered nurses with academic credentials (two professors, two associate professors and one assistant professor), skilled in moderating focus groups.

## Results

Thirty-two students participated in the study; 19 were full-time students, while 13 were part-time students. The students were between 20 and 40 years old. Five students were male, and the others were female.

Two main categories were identified from the data analysis. (1) Facilitator as a catalyst for reflection and (2) a process towards increased awareness. Table 2 displays the main categories with their associated subcategories.

**Table 2:** Main categories with subcategories

Main categories	Subcategories
Facilitator as a catalyst for reflection	Balancing safety and challenges Providing constructive feedback Facilitating the discovery of coherence and alternative options Providing structure as a prerequisite for dialogue
A process towards increased awareness	Advancing in how to provide and receive feedback Proceeding action readiness Progressing through self-discovery

## Facilitator as a catalyst for reflection

The first main category emphasizes the facilitator’s multifaceted and important role in initiating and guiding the students’ reflection process in the debriefing. Four sub-categories elucidate this main category: balancing safety and challenges, providing constructive feedback, facilitating the discovery of coherence and alternative options, and providing structure as a prerequisite for dialogue.

### Balancing safety and challenges

The results show that the students expressed that their learning process was strengthened by the balance between feeling safe and being challenged. The students described the importance of the facilitator’s ability to balance when to ask questions and when to provide an explanation so that the students did not feel too pushed if not able to answer: “If the facilitator asked you a question and you felt you couldn’t answer, they explained instead of putting real pressure on you” (FGI 1)*.* The students described this as promoting their safety and self-confidence. At the same time, they also valued when the facilitators challenged them to put into words and to elaborate thoughts as one student expressed: “When you said something, they [the facilitators] could ask: Can you elaborate a bit more? What do you mean by that? They challenged us to elaborate more and to explain what we were thinking” (FGI 3). Further, the students experienced that the facilitator had a deliberate way of posing questions, which challenged students to justify their actions and their ability to reflect. One participant uttered: “Nice that they [the facilitators] asked us to justify what we had done…that we could try and reflect on what we had done. That it wasn’t just like ‘this you did well and here you made a mistake’, instead they asked: ‘but why did you do that?’ because then we consider that we should not just act without having a reason to do so” (FGI 1).

### Providing constructive feedback

The students described how the facilitators provided feedback by starting with positive aspects before moving to areas that could have been performed better or differently, causing the students to feel less stressed and not embarrassed: “They [the facilitators] were good at starting with the positives…and constructive feedback was given in a very nice way, so you didn’t feel stupid” (FGI 4)*.* In contrast to how the facilitators emphasized what went well during the simulated scenario, the students described how they quickly focused on mistakes. Hence, support from the facilitator to provide a positive perspective on their own and fellow students’ performances was emphasized. Although the students described it as crucial that the facilitator started with positive feedback, they also expressed the importance of feedback that addressed whether they had misunderstood or acted incorrectly and the potential for improvement. The students perceived the facilitator as a reliable source of feedback. It was reassuring to receive feedback from the facilitator on their thoughts, assessments and actions that had been conducted or observed, as the students did not always fully trust their own judgement: “You’re not quite sure if you’re doing what you’re supposed to do or if you interpreted things correctly. Am I in the right place at the right time? Do I understand what this is? In fact, it’s reassuring to get feedback from the facilitator to understand where you are on the trail” (FGI 2)*.* Feedback from and discussions with fellow students were perceived as helpful. However, there were concerns regarding fellow students’ ability to assess and understand the wholeness of the situation. Hence, they needed feedback from the facilitator to validate fellow students’ feedback: “… if we start to discuss in the group, then we might come up with many points of view, but in a way, what is the right one? … very good that they [the facilitators] took part in the debriefing to be the clarifying factor …” (FGI 1).

### Facilitating the discovery of coherence and alternative options

The students described how the facilitator encouraged them to discuss and reflect on different viewpoints on performing alternative actions. They expressed that the facilitator, by combining theory and experiential knowledge in their teaching, made them better understand the coherence and variations in experienced simulated scenarios. The students described a development in their awareness of discovering alternative solutions compared with previously seeing just one solution: “…it was said that there are so many different points of view and ways of doing it that I feel that the knowledge sits better; now it's not just one solution anymore, now I actually have many different solutions” (FGI 1).

The students expressed that thinking about and discussing what they could have done differently and considering possible action options was educational. One student who had felt insecure about what she was allowed to communicate to the patient’s relatives appreciated when the facilitator shared her own experiences as a nurse in clinical practice: “The facilitator came forward with a situation that she had experienced, a very unpleasant situation (related to the duty of confidentiality), so you have to be very careful about what you say and how you say it.” (FGI 3)*.* The fact that the facilitator used their own experiences was described as instructive and helpful in discovering connections and possible alternative actions, as the students lacked experience in similar situations.

The participants perceived it valuable to ask the facilitator during the debriefing how the simulated scenario was supposed to develop and what other options for action there were. The students expressed that the facilitator’s request increased their understanding of various patient situations and action alternatives in the simulated scenario. One stated: “Then we could ask the facilitator how this could have developed, and what we could have done further, so you then could acquire a certain picture of it in your head.” (FGI 1). The students found it inspiring when their prior knowledge and experience acquired during the simulated scenario helped them link previous knowledge and possible actions.

### Providing structure as a prerequisite for dialogue

The students expressed that the facilitators played a critical role in achieving valuable discussion and reflection and providing structure in the debriefing. A significant part of this role concerned supporting the students in focusing on the learning objectives and extracting meaningful learning from the simulated scenario. The facilitators managed the debriefing in a way that created a beneficial condition for reflection and learning. The students appreciated that the facilitators ensured everyone had time to talk, provided questions and views, and guided them to emphasize key themes. If the conversation stopped, then the students appreciated the facilitator’s intervention: “… then it was good to have someone who could point out and draw the answers out of us … for sometimes it just stops and then, yes, then nothing happens” (FGI 1).

While most students stated that the facilitators managed the time well and ensured they had sufficient time to reflect on their actions, some students expressed a different experience. Some felt that the facilitators spent too much time talking or unnecessarily stretching the time, and they also wanted a shorter and more structured debriefing. A prolonged debriefing could lead to unnecessary repetition, resulting in less concentrated or restless students. Other students had a more nuanced view regarding the facilitators’ time management of the debriefing:

“...the debriefings were a bit long, but I also think it was good because then you didn't feel pressed for time when asking questions. Because many things were clarified during the debriefing, there were many questions, there were many points of view, but sometimes I think that the facilitator unnecessarily delayed it, and then someone became unconcentrated.” (FGI 2).

## A process towards increased awareness

The second main category encompasses the students’ experience of the facilitator’s role in guiding them to join parts together and see the wholeness in what was simulated. The debriefing could be viewed as the students’ process of acquiring new insight into their professional development. This process is described through the following three sub-categories: advancing in how to provide and receive feedback, proceeding action readiness and progressing through self-discovery.

### Advancing in how to provide and receive feedback

The students said they initially found it challenging to know what they were supposed to provide and receive feedback on, as the facilitator did not explicitly address this issue. However, the observation sheets they received from the facilitator provided some structure and security: “… those sheets had a lot to say to be able to give feedback because without them I think you would be sitting there as a question mark…” (FGI 4). As they developed greater confidence in what to provide feedback on, the students also managed to provide feedback beyond assigned points. One student said it like this: “…It’s fine that we are given particular points to observe, but I looked a little at the other points as well because one might see things that the others don’t see, or you look at it differently” (FGI 2).

How to provide feedback to peers was described as difficult because large groups or unknown fellow students could lead to a feeling of insecurity and result in vague or undivided positive feedback. Several students found it challenging to provide negative feedback to peers, especially without any guidance from the facilitator on this issue in the first debriefings. They described a demanding balancing act between giving feedback on mistakes or inappropriate actions and at the same time taking care of their fellow students’ feelings: “I’m so afraid of saying something wrong, you don’t know how people will take it. Some can take it personally.” (FGI 3).

The students developed a greater awareness of and confidence in providing and receiving feedback after several debriefing experiences. They described becoming more confident with peers, which influenced their way of providing feedback. The students dared to be more honest when participating in small and larger groups and with unknown fellow students. One student expressed it like this: “We got tougher and tougher after each scenario to not only give positive feedback, tougher to say that ‘yeah, you’re not checking that bracelet, are you?’” (FGI 4).

The students’ increased awareness of providing and receiving feedback also opened the door for alternative feedback methods, such as using humour or recognition. Using oneself as an example could seem harmless and take care of fellow students’ feelings at the same time that mistakes were addressed: “In a slightly funny way…I see you made the same mistakes as I did; it turned out a bit like that, and then it was okay in a way.” (FGI 4).

The findings show that providing a safe learning environment is essential to ensuring each student’s development in providing and receiving feedback.

### Proceeding action readiness

The students expressed that the reflection in the debriefing guided by the facilitator increased their awareness of their practice. By evaluating and assessing their actions with guidance from the facilitator, they increased their understanding of what influenced their actions and how their actions, or lack of actions, could affect the patient’s health. One explained: “And then I experienced the importance of planning, in connection with the patient saying he was nauseated, and I didn’t think anything more about it, and then he throws up… if I had thought a little longer when the patient said he was nauseous, then I should have had a kidney dish ready… I will remember that from now on” (FGI 3). The participants described that reflecting on their experiences of unforeseen situations, as this last quote refers to, contributed to increased predictability and the development of appropriate strategies.

The students greatly benefited from reflecting on their own and peers’ mistakes. They experienced that through making mistakes, they became aware of their preconceptions about their nursing skills and that there was not always a relationship between what they believed they had mastered in advance and what they experienced mastering: “… I thought I knew cardiac arrest and knew how to handle it, and then I get there and have no idea, and then it was good to be able to talk about it afterwards, what did I do wrong and things like that” (FGI 1).

Students experienced that they had better understood what clinical practice required through the facilitator’s guidance in the debriefing. Students expressed that the facilitation made them more aware of the importance of priorities and order of action. This involved evaluating the situation by separating the important issues from the unimportant. One student explained:

“If we come in and see a patient having difficulty breathing, we have learned that we have to act, put in the nasal glasses, put on oxygen. Earlier, we stood there and stroked the patient’s hand, and then nothing more happened. Now we have become more aware of our actions. When you come in to see a patient now, you don’t just stand there and pamper....” (FGI 4).

The students described how the facilitator’s encouragement of thinking and discussion of the course of action in the debriefing helped them discover a new understanding of the situation. They described an experience of faith in one’s mastery and better preparedness for similar future situations.

### Progressing through self-discovery

The students expressed how the facilitator’s guided reflection in the debriefing affected their understanding of themselves. They became more aware of the reasoning behind their choices and actions by asking themselves questions. For example, they discovered how stress could affect what they remembered from the simulation scenario, impacting their learning. In this context, several emphasized the video recordings as helpful in becoming aware of their own performance, reactions and attitudes. One student explained:

“I stood there fiddling with my hair; it wasn’t appropriate. Watching the video lowered the stress so that you can more easily see what you should have done differently... because then you can reflect on what was good and what was not.” (FGI 3).

Students felt that watching themselves on the video provided a more differentiated view of their performance. They expressed that they could underestimate their knowledge and skills and that watching themselves “from the outside” could change their view of an assessment of their performance: “… you think about it afterwards, and then you see it, and then just yes, this went well, somehow” (FGI 3).

Putting into words and speaking aloud about one’s actions and feelings could increase awareness of one’s knowledge and skills. One student expressed*:*

“...it’s different to talk about it out loud...I’ve learned it, but it’s far back of my mind, but when you talk about it out loud, it’s easier to remember, to think back that, oh yes, it was like that. You bring forth different points of view....” (FGI 1).

The students described the facilitator’s guidance of the conversation and discussion in the debriefing as pivotal in linking previous knowledge to the actions performed. The debriefing contributed to increased self-insight through awareness of what they had done, how they had acted and the rationale behind their actions. Facilitation in seeing the situation and one’s actions from different angles was described as contributing to being able to view one’s actions in a bigger picture.

## Discussion

The present study aimed to explore nursing students’ perspectives on the post-simulation debriefing. The results showed that the facilitators had a multifaceted and vital role in initiating and guiding the students’ reflection process in the debriefing. The facilitator was also pivotal in the students’ guidance in putting pieces together and seeing the wholeness in what was simulated. The students viewed the debriefing as a reflective process of acquiring new insight into their professional development.

In the present study, the students expressed that their learning process was strengthened by the balance between feeling safe and being challenged by the facilitators, who provided positive feedback and did not humiliate them by starting with positive aspects before moving to what could have been performed better or differently. This is consistent with findings that students experienced the learning environment as supportive and safe when faculty provided positive feedback and constructive criticism [26, 39]. Our results contrast with the study by Ko and Choi [37], which showed that the students sometimes were emotionally injured upon receiving negative feedback from the facilitator during the debriefing. This made the students sensitive to interpersonal relationships with team members or professors during the simulation. One explanation of the variations in the findings might be that SBL will be influenced by the norms, values and beliefs held by the participants interacting in SBL [51]. A recent study by Turner et al. [52] found that how the facilitator approached the students in the simulation and provided verbal feedback, and their non-verbal reactions influenced the students’ sense of psychological safety. In SBL, a psychologically safe environment offers the opportunity to learn from mistakes through constructive feedback without criticism, providing ample time to solve the challenge and receive immediate support without penalty [53]. Nurse educators should consider optimal simulation design features to increase psychological safety because it [54] plays an essential role in achieving learning outcomes of SBL [55]. In the present study, the students emphasized the importance of feedback which addressed incorrect actions to improve. This is consistent with other findings by Ko and Choi [37], which showed that the students appreciated the fact that the facilitator said it was acceptable to make a mistake and made the students comfortable.

Participants described how the facilitator encouraged them to reflect on and discuss different viewpoints on performing alternative actions during the conversations. The students experienced that previously developed knowledge and learning acquired during the simulated scenarios had helped them discover the relationship between their knowledge and possible actions. Similarly, Nash et al. [41] found that the post-simulation debriefing helped the students assimilate what they had learned, connect it to previous learning and plan how it might be applied in future practice situations.

Findings indicated that the students appreciated when the facilitator shared their patient experiences. Sharing experiences was also helpful for discovering relationships and possible actions. This finding echoes the results in a previous study by Fey et al. [37, 39], which found that the facilitator used several techniques, e.g. using their own experience, to create a positive learning environment and to encourage students’ learning and reflection. Our findings showed that the facilitator’s role was critical in achieving a valuable reflective conversation and providing a structure in the debriefing. Fey et al. [39] found that the facilitator used a beneficial structure of the debriefing for the students by moving through the phases of addressing emotions, reflecting and summarizing.

In contrast, findings by Ko and Choi [37] show that the nursing students experienced that the facilitators used very different methods and that a lack of standardized education policies made the participants uncomfortable. One explanation of the contrasting findings might be that differences in facilitator skills will impact the debriefing methods used in SBL and thereby entail variation in students’ experiences with the facilitator’s role [56]. The HSSOBP™ The Debriefing Process [17] noted that debriefing facilitators require training in structure and techniques. Similarly, a review by Hall and Tori [34] found that debriefing facilitators should have formal training and need to practice in simulated environments. Debriefing skills should be refined through ongoing educational activities, peer assessments and self-education.

Some students participating in this study felt that the facilitators spent too much time talking or unnecessarily stretching the time and wished for a shorter and more structured debriefing. Der Sahakian et al. [57] recommend that a facilitator respect any predefined schedule regarding the duration of the debriefing but also maintain control over who speaks and for how long. The authors suggest obtaining anonymous feedback from the learners and peers about the simulation session that can enhance the continuous development of the instructors and promote each facilitator’s reflection on their own performance as an instructor.

The students in the present study described that they initially found it challenging to know how to receive feedback from peers and what feedback to provide. However, the participants described that these skills were developed throughout the SBL. One explanation of these findings might be that the nursing students felt uncertain because they had not developed peer feedback skills previously in their education. This explanation is supported by a recent review of nursing students’ experience and perception of feedback, which found several challenges faced in using peer feedback, such as feeling underqualified to give feedback and causing initial unease among students [58]. The students asked to include more training and guidance in the nursing curriculum. Skill in mentoring peers has been demonstrated as a crucial professional competence to achieve during nursing education [59]. SBL might support nursing students in developing this professional competence in a group of peers who can support, learn with and from each other.

Findings suggest that making mistakes created awareness of preconceptions regarding one’s own nursing skills and the potential mismatch between perceived mastery and actual execution of skills. Tai et al. [60] propose that developing students’ evaluative judgement should be a goal of higher education, to enable students to improve their work, clarify their learning goals and be aware of what they need to master. They define evaluative judgement as “the capability to make decisions about the quality of work of self and others” [60, p. 471]. The students must gain an understanding of how to make evaluative judgements, so that they may operate independently on future occasions, taking into account all forms of feedback comments, without explicit feedback from a facilitator or peers [60]. SBL might contribute to preparing students for professional practice by developing evaluative skills that they can use to identify what is needed to demonstrate high-quality work in professional settings.

The findings in the present study revealed that the students’ reflections in the debriefing affected their understanding of themselves. They learned about themselves and became more aware of the reasoning behind their own choices and actions by questioning how they managed the various patient situations in the simulated scenarios. The students’ reflections regarding self-discovery and self-insight align with the description of reflection by Ekebergh [61]. Ekebergh [61] proposes that reflection is about self-insight turning towards oneself to discover oneself; that is, thoughts and feelings are directed at one’s consciousness. The process of reflection can be understood as a development process in understanding [62]. Ekebergh [62] states that we can put old truths and self-evident at stake through an investigative and questioning attitude.

Students found viewing the video playback helpful in becoming aware of their performances, reactions and attitudes, as well as providing a differentiated view of their performances. They could underestimate their knowledge and skills, and watching themselves “from the outside” could change their self-assessment of their performance. Similarly, Reierson et al. [63] found that watching their performance on video was somewhat embarrassing but helped the nursing students to see what they were very aware of, how they communicated and their attitude toward patients. The students in Reierson et al. [63] who watched the video recordings before the joint debriefing were more active in the debriefing compared with students who did not watch the video of their performance.

## Limitations

One limitation of this study is that, due to practical considerations, the participants were not contacted to approve the transcripts of the FGIs and the analysed data. The sample of students recruited from one university attending the same SBL course may have resulted in more one-sided data than if the study had included students from more than one university. That only five males participated in the study may have influenced the findings. However, most of the nursing students were women. The different compositions of the moderator teams might have resulted in varying amounts of rich information from the different FGIs. Therefore, the number of citations reproduced from each FGI might be unbalanced. Since the Norwegian language was used in the SBL and the FGIs, and the involved reflections have been translated into English, slight differences in linguistic nuances may exist when the data are collected in Norwegian but written in English.

### Trustworthiness

We sought to enhance the trustworthiness as suggested by Lincoln and Guba (1985). To enhance the credibility of the findings, all authors participated in all stages of the analysis, agreeing on the key patterns identified and engaging in all phases of writing the article. We also sought to reflect upon our pre-understandings. Three authors (IÅR, AH, HS) were not part of the data collection and provided an external perspective of the study. We suggest that the findings will interest the simulation community, as debriefing is a crucial aspect of SBL. We contribute to an in-depth knowledge of how nursing students perceive the post-simulation debriefing. As such, we propose that the findings can guide facilitators on how SBL might contribute to professional development in nursing education.

## Conclusions

This study provides knowledge to facilitators regarding nursing students’ perspectives on facilitating reflection and learning during debriefing discussions. The facilitator’s multifaceted role in guiding students’ reflections and process of acquiring new insight into their professional development was identified as critical to learning during debriefing.

This study has shown that facilitation education should emphasize how students’ reflection process can be guided in the debriefing. The debriefing might also be critical to students’ development of evaluative judgement.

### Supplementary Information

Additional file 1

## Data Availability

Not applicable. The data supporting this study’s findings are not available due to ethical approval for the study that requires the transcriptions of the FGIs to be kept in locked files, accessible only by the authors.

## Abbreviations

FGIs: Focus group interviews
SBL: Simulation-based learning
