# Supplementary material for: Post-simulation debriefing as a stepping stone to self-reflection and increased awareness — a qualitative study
Source: Adv Simul (Lond). 2024 Aug 13;9:33. doi: 10.1186/s41077-024-00306-2 (PMC11320937; doi:10.1186/s41077-024-00306-2)
Supplement: Supplementary file 1 — Additional file 1 [file 41077_2024_306_MOESM1_ESM.docx]

**Additional file 1** Interview guide (translated from Norwegian to English)

1. How did you experience the usefulness of the debriefing concerning learning outcomes for the simulation? (Explain, if necessary, what is meant by debriefing)

1. What importance do you think the facilitator had during the debriefing?
2. How did the feedback from the facilitator contribute constructively concerning what you could improve?
3. How did you feel about being able to speak with your questions and comments?
4. Can you say something about the time set aside for debriefing to be evenly distributed between you and the facilitator?
5. How do you assess that the facilitator's role in the debriefing contributed to you being able to achieve the learning outcomes?

2. In what way can debriefing help ensure that what you learned during the simulation can be applied in practice?
